# Risk Stratification of Pancreatic Neuroendocrine Neoplasms Based on Clinical, Pathological, and Molecular Characteristics

**DOI:** 10.3390/jcm11247456

**Published:** 2022-12-15

**Authors:** Jin Ho Choi, Woo Hyun Paik

**Affiliations:** Department of Internal Medicine, Seoul National University Hospital, Seoul 03080, Republic of Korea

**Keywords:** pancreatic neuroendocrine tumor, prognostic factors, clinicopathological factors, genetic factors, epigenetic factors

## Abstract

Pancreatic neuroendocrine neoplasms consist of heterogeneous diseases. Depending on the novel features detected by various modern technologies, their classification and related prognosis predictions continue to change and develop. The role of traditional clinicopathological prognostic factors, including classification systems, is also being refined, and several attempts have been made to predict a more accurate prognosis through novel serum biomarkers, genetic factors, and epigenetic factors that have been identified through various state-of-the-art molecular techniques with multiomics sequencing. In this review article, the latest research results including the traditional approach to prognostic factors and recent advanced strategies for risk stratification of pancreatic neuroendocrine neoplasms based on clinical, pathological, and molecular characteristics are summarized. Predicting prognosis through multi-factorial assessments seems to be more efficacious, and prognostic factors through noninvasive methods are expected to develop further advances in liquid biopsy in the future.

## 1. Introduction

Accounting for approximately 5% of all pancreatic tumors, pancreatic neuroendocrine neoplasms (pNENs) are the second most common mass [1,2]. For newly diagnosed pNENs, it is important to perform risk stratification to estimate the prognosis. There are still many obstacles to overcome in achieving accurate prognostic predictions, and more sophisticated prognostic predictions have been developed with the recent development of next-generation sequencing and other high-throughput advanced molecular techniques. Classical prognostic prediction is conducted on the basis of clinicopathological prognostic factors, including disease stage, several serum biomarkers, and the WHO grading system. Classification of pNEN contributes the most to prognosis, and recent classification of pNENs is according to the 2019 WHO classification of tumors of the digestive system on the basis of differentiation and cell proliferation [3]. pNENs are largely classified into two groups: well-differentiated pancreatic neuroendocrine tumors (WD-pNETs) and pancreatic neuroendocrine carcinoma (pNEC) [1,3]. WD-pNETs are usually indolent compared to pNEC and have a better prognosis; however, prognosis prediction is very important to determine appropriate management, as these tumors exhibit a heterogeneous extent of disease [1,4,5]. Molecular prognostic factors, including numerous genetic and epigenetic prognostic factors, have been reported in association with prognosis of pNENs, and remarkable advances were achieved in multifactorial prediction models, including molecular biomarkers and multiomics sequencing in accordance with them. In addition, considerable advances were achieved, especially in liquid biopsy for pNENs. In this narrative review, the traditional and recent advanced strategies for pNEN risk stratification based on clinical, pathological, and molecular characteristics are comprehensively reviewed.

## 2. Main Body

### 2.1. Classification of pNEN

Currently, neuroendocrine neoplasms are classified according to World Health Organization (WHO) classification (Table 1), which is most commonly used. Since there are differences in mutational profiles between pNENs and other gastroenteropancreatic neuroendocrine neoplasms (GEP-NENs), it is considered as a different disease entity, and prognosis prediction in pNENs has an independent aspect [6]. The American Joint Committee on Cancer (AJCC) and European Neuroendocrine Tumor Society (ENETS) staging criteria have been most commonly used to predict prognosis of pNENs [7,8,9,10]. One of the limitations of the previously proposed classifications was that grade 3 pNENs were very heterogeneous, which render prognosis prediction difficult, and WHO 2017 classification suggested the reclassification of grade 3 pNETs from poorly differentiated pNEN (PD-pNEN) to grade 3 well-differentiated pNET (WD-pNET). WD-pNETs were subdivided into low-grade (G1), intermediate-grade (G2), and high-grade (G3) tumors, and PD-pNEN includes neuroendocrine carcinoma (NEC) only, which was subdivided into large-cell or small-cell subgroups according to cell morphology. Large cell NEC harbors alterations in BRAF, MYC, and ARID1A more frequently in comparison with small cell NEC. Instead, mutations in the MAP3K1 gene were enriched in small cell NEC. In addition, small cell gastroenteropancreatic NEC had a significantly worse prognosis than large cell gastroenteropancreatic NEC within the NEC group [11]. Until now, the results from the majority of previous studies for the therapeutic management of high-grade NENs should be interpreted with caution because those studies contained mixed high-grade WD-pNET and NEC. Treatment for high-grade pNENs has not yet been standardized because there is a lack of evidence for the optimal treatment of high-grade pNENs according to the latest classification [12]. The WHO 2019 classification was recently introduced for GEP-NENs, and there was no significant change in the pNEN classification [3]. 

Due to tumoral heterogeneity and necrosis, the differentiation between grade 3 WD-pNET and pNEC in pathological morphology is quite equivocal [13]. For the Ki-67 index, grade 3 WD-pNET is known to have a median of 30% (range 20–50%), and pNEC has a higher median value of 80% (range up to 100%) [14]. The molecular features of pNEC are considerably different from WD-pNET, and more extensive research results have been reported regarding molecular biological properties in pNET cases [15]. Inactivation mutations in DAXX/ATRX and MEN1 are known to be exclusively found in pNET, and other mutations in the PI3K/mTOR signaling pathway, such as phosphatase and tensin homolog [PTEN], DEPDC5, and PIK3CA, are found in WD-pNET [4,16,17]. In addition, most pNETs demonstrate hemizygous loss of specific chromosomes [15]. In contrast to TP53, RB1, and/or CCNE1/MYC amplification alternations are commonly found in pNEC [15]. According to a recently reported genomic deep analysis, including whole-genome/exome sequencing, transcriptome sequencing, DNA methylation assays, and/or ATAC-seq for 115 patients, pNEC showed moderately different genomic features from nonpancreatic NEC, which have more structural variations and nonsynonymous mutations [15]. Furthermore, pNEC can be classified into two subgroups, such as ductal and acinar types, based on genomic alterations. Most ductal-type pNECs show an RB1 protein loss and TP53 mutations, and generally have CpG island methylator phenotypes (CIMP). The acinar-type pNEC featured altered Wnt signaling and cyclin-dependent kinase inhibitor 2A (CDKN2A) alterations [15]. Epigenetic aberrations also play a significant role in pNEC; in particular, transcription factors, such as the SOX2 gene involved in the development of neuroendocrine tissue, were overexpressed in pNEC through promoter region hypermethylation [15,18,19].

### 2.2. Prediction of Prognosis: Risk Stratification in pNEN

Predictions of prognosis could be determined based on various aspects, including patient factors, disease features, and treatments. This review summarizes the extensive evidence for prognostic predictions of pNENs, rather than predictions of the response to specific treatment. This review is for the purpose of conveying information as a narrative review, not a clinical guideline. The results of each study should be conservatively interpreted.

#### 2.2.1. Clinicopathological Prognostic Factors

A wide variety of clinical and pathological factors have been used to evaluate pNEN prognosis. Clinical factors include epidemiological factors, medical conditions, and various test results, such as blood tests and medical imaging. The predictive method using these factors has been developed to have better efficacy by modifying the typical cutoff value. For example, a 10% threshold value for Ki-67 index appears to be more successful in predicting associations with mortality compared with the current grading criteria [20]. Regarding tumor size, it was previously considered safe to be less than 2 cm, but recent studies have shown malignant potential even in tumors less than 2 cm, and the size definition of small pNEN continues to change [21]. In line with this, de novo prognostic factors are also under way. Furthermore, due to the development of new technologies such as digital pathology, liquid biopsy, and artificial intelligence, including deep learning, these factors are continuously and significantly used.

##### Clinical Prognostic Factors

1) Tumor-Node-Metastasis (TNM) Stage

The representative prognostic factor of pNENs is the TNM stage by the AJCC 8th edition and ENETS (Table 2) [7,9,22,23,24,25,26,27,28,29,30,31,32,33]. The ENETS TNM staging system for PNETs better delineates features that are different from pancreatic exocrine carcinoma, and it provides better predictive power compared to the AJCC 7th edition staging system; however, discrimination between stage IIIA and IIIB is limited [34,35,36]. The modified ENETS TNM staging was introduced and showed better outcomes with maintenance of the T stage of the ENETS staging system and the adoption of the staging definition of the AJCC system [37]. Moreover, further modification of the N stage showed better prognostic value and proportional distribution of pNENs [38]. Consequently, in the AJCC 8th edition TNM staging system, the definition of the T stage was revised as ENETS systems, and it reported a better prognostic predictive value for PNETs [39].

2) Other Clinical Factors

Further clinical factors are shown in Table 3. Age is one of the well-known prognostic factors for overall survival (OS), and the age criteria vary from 55 to 65 years old; older age is associated with a worse OS, including worse survival after surgical resection [26,32,40]. Functional pNENs are also associated with a longer OS than nonfunctional pNENs [22,26,32]. Patients who were deemed candidates for surgical resection for pNENs showed better outcomes, but the absence of macroscopic radical surgery was associated with recurrence after surgical resection and was associated with poor OS [25,27,40,41].

3) 68Gallium Positron Emission Tomography/Computed Tomography (^68^Ga-DOTATOC PET/CT)

The ^68^Ga-DOTATOC PET/CT is usually used to diagnose pNENs with somatostatin receptor overexpression, and it was used to estimate for peptide receptor radionuclide therapy feasibility [42]. ^68^Ga-DOTATOC PET/CT distinguishes between heterogeneous clinical phenotypes of pNENs, addresses the limitations of the histopathological grading system, and allows precise staging assessment for appropriate management strategies by physicians [42,43]. Furthermore, ^68^Ga-DOTANOC predicts pNENs prognosis, and a maximum standardized uptake value of less than 37.8 is associated with disease progression in patients with G1 and G2 pNET, and higher ^68^Ga-DOTATATE total volume is associated with decrement in progression-free survival (PFS) and increment in disease-specific mortality [44,45]. Despite ^68^Ga-DOTATOC PET/CT showing clear benefits for the detection and staging of pNEN, it does not make ^18^F-FDG PET/CT redundant [42]. A combined ^68^Ga-DOTATOC PET/CT and ^18^F-FDG PET/CT surpass the limit of WHO grading and TNM staging in prognostic predictions of pNEN [43].

4) Serum Biomarkers

Various laboratory tests have also provided information through serum biomarkers to predict prognosis (Table 3). Numerous researchers have tried to find a serum biomarker, but they have been unsuccessful, mainly due to the heterogenous nature of pNENs [46]. Serum biomarkers have mainly focused on measurements of secretory products, which result in insufficient ideal diagnostic sensitivity, specificity, and prediction power for prognosis [46,47]. Nevertheless, their clinical importance still remains in consideration of non-invasiveness and being repeatable in nature.

In functional pNENs, each related hormone, such as insulin, glucagon, vasoactive intestinal polypeptide, gastrin, and somatostatin, is also useful to predict recurrence and treatment response, aside from its utility in diagnosis [47]. One of the most reliable and practical general serum biomarkers is plasma chromogranin A (CgA), which is an acid glycoprotein present in the secretory dense core granules of most neuroendocrine cells. Moreover, elevations in CgA can be observed in both functional and nonfunctional pNENs [47]. Serum CgA level has been associated with the Ki-67 index, WHO classification, TNM stage, and treatment response [47,48,49,50]. Increased CgA levels are predictive of disease recurrence during 9–12 months after surgery, and an early decrease in CgA after treatment is correlated with prolonged survival [47]. One study reported that a two-fold rise in the upper limit of CgA was associated with a shorter OS [51]. Recently, a set of circulating miRNAs showed an increase in diagnostic performance of CgA in pNENs, even with low CgA in patients with metastatic pNENs [52]. Neuron-specific enolase (NSE), a diagnostic and prognostic marker, was elevated in 30–50% of patients with pNENs, particularly in those with PD-pNETs, and its level was associated with OS and treatment response to everolimus [47,53,54]. CgA is known to have increased sensitivity than NSE, and the combined monitoring of CgA and NSE is more accurate in predicting prognosis and disease progression [55,56]. Pancreastatin is a fragment of the CgA molecule, and it is a good marker for pNENs, with better efficacy than CgA. Higher pancreastatin levels are associated with significantly worse PFS and OS, and negative pancreastatin responses to somatostatin analogs are associated with poor OS [57,58]. In WD-pNETs, a higher serum albumin-to-alkaline phosphatase ratio showed more favorable prognosis [59]. Preoperative main pancreatic duct dilatation and neutrophil-to-lymphocyte ratio in pNENs were independent predictors of OS and disease-free survival (DFS) for pNENs of the pancreatic head [60]. Overall, despite the controversial issue, CgA has been found to be the most practical and useful general serum biomarker in pNENs, with relatively good sensitivity and specificity, but a prognostic value of CgA for pNENs has not been completely validated due to a lack of reliable evidence [47]. In addition, it should be considered, for interpreting the level of CgA, that it might be influenced by various factors, including the type of assays, impaired renal function, atrophic gastritis, and steroid and proton-pump inhibitor treatment [47].

pNENs are associated with extensive neoangiogenesis compared with other tumors; hence, angiogenic factors have also been suggested as potential biomarkers [61,62]. Vascular endothelial growth factor (VEGF), a key angiogenic factor, which has been largely studied for its prognostic role as a possible therapeutic target, is also associated with locoregional tumor spread and tumor progression, and can be considered as a prognostic marker and therapy control in patients with pNENs [63,64]. Placental growth factor, a VEGFA homolog, which is expressed primarily in the stroma, is positively correlated with advanced tumor grading and is negatively correlated with reduced survival [65]. Elevated serum levels of angiopoietin-2 were observed in patients with advanced pNENs [62,66,67,68]. Overall, angiogenic factors provide information on the state of vascularization of the tumor tissue and/or the tumor microenvironment of pNENs, unlike the other serum biomarkers from secretory products. Along with promising results, angiogenetic factors will be a representative type of biomarker and therapeutic target for treatment of pNENs in the future.

##### Pathological Prognostic Factors

1) WHO Grading System: Differentiation and Cell Proliferation

The WHO classifications showed significant value in predicting the prognosis of pNENs, including tumor grade and differentiation. The majority of the changes in the classification for pNENs occurred in 2017, and molecular biological evidence was solidified while maintaining the overall classification in the 2019 WHO grading system [3,10]. The reported 5-year survival rates of G1, G2, and G3 are 75%, 62%, and 7%, respectively [69]. The WHO classification presents relatively simple and excellent criteria that provide intuitive information regarding the overall prognosis of pNENs [10].

In terms of tumor differentiation, pNECs are discriminated based on severe cellular atypia, high-proliferative rates, and focal to extensive necrosis, and poor differentiation is associated with poor survival outcomes [25,33,70]. Tumor grade is classified based on cell proliferation according to the mitotic count or Ki-67 index, and high cell proliferation is associated with poor clinical outcomes. Mitotic rates are to be expressed as the number of mitoses within 10 high-power fields at 40× magnification, which are manually counted by pathologists [3]. The Ki-67 protein is associated with cell proliferation, and its expression is limited to active phases of the cell cycle [71]. Using immunohistochemistry, Ki-67 protein can be detected, and guidelines suggest that the Ki-67 proliferative index for NENs should be assessed within hotspot areas consisting of 500–2000 tumor cells [3,10]. A lower Ki-67 index in pNENs was associated with better OS [25,27,41]. The Ki-67 index is also a significant predictive factor for DFS and OS in resected pNENs [21,22,23,72]. According to a large study with 505 pNEN patients who underwent R0 resection, high-grade (Ki-67 > 20%) tumors were associated with a 7.8-fold increase in risk of recurrence and a two-fold increase in risk of death compared with low-grade (Ki-67 ≤ 2%) tumors [22]. Each previous study arbitrarily sets the cutoff value of Ki-67 and varies from 2% to 20%; however, consistent results have been reported and that a higher Ki-67 is associated with a worse prognosis. Ki-67 is a very efficient tool for measuring cell proliferation; however, concerns regarding the method of measurement have been outlined. The size of hotspots has been determined based on intuition rather than solid evidence [73]. The type of anti-Ki67 antibodies, specimen type, and the size of hotspots affect the Ki-67 index. Increasing the hotspot size decreases the Ki-67 index, including the standardization of antibody clone selection and hot spot size; moreover, a complete consensus has not been reached in terms of antibody clone selection and hotspot size for grading pNENs [74]. Additionally, a systematic review with meta-analysis reported that digital image analysis appears to offer pathologists greater reliability and reproducibility than manual counting for grading pNENs, and digital image analysis-based methods for Ki-67 counting will be widely applied in the near future [75].

Recently, endoscopic ultrasound (EUS)-guided fine-needle biopsy (EUS-FNB) has become one of the most common methods to obtain tissue from pNENs to estimate the Ki-67 index, except for surgical resection. It is possible to acquire the tissue of pNENs to analyze the Ki-67 index through EUS-FNB in general [21,76]. However, the estimated Ki-67 index on EUS-FNB samples may not be representative of the entire tumor, as an EUS-FNB specimen is only a fraction of the tumor. It was especially underestimated in grade 2 pNENs on cell block material, and the hotspots in the EUS-FNB specimen should be defined as more than 1000 cells to reach better correlation with surgical specimens [77]. Recently, a study reported that double Ki-67 and synaptophysin immunolabeling enables a more accurate measurement of the number of proliferating tumor cells compared with a single Ki-67 immunolabeling in pNENs from EUS-FNB specimens [78].

2) Other Pathological Prognostic Factors

The ratio between the number of metastatic lymph nodes and that of the examined lymph nodes is defined as the lymph node ratio, and it has been determined to be a strong prognostic factor for DFS and OS in resected pNENs [72]. Moreover, a lymph node ratio ≥ 0.5 is independently associated with worse disease-specific survival [79]. Despite the clinical importance of lymph node metastasis as a prognostic factor for survival and recurrence after surgery, the definite therapeutic role of regional lymphadenectomy in patients who underwent surgical resection for pNENs remains controversial according to previous studies [80,81]. Even patients who underwent surgical resection with lymphadenectomy for a median of 9 regional lymph nodes did not show therapeutic benefits [80]. Another retrospective study suggested regional lymphadenectomy should be performed in grade 2 or grade 3 patients, but should not be mandatory in grade 1 tumors with a size of less than 4 cm [82]. In the future, it seems necessary to find more robust and high-level evidence, and to present a proper indication for lymphadenectomy.

Neuroinvasion or vascular invasion is associated with poor outcomes [33]. pNENs with peritumoral infiltrating and stromal desmoplastic reactions have been associated with poor clinical outcomes [33]. To predict recurrence in surgically resected nonfunctional pNENs, tumor-associated macrophages and a high CD68 scores, as a measurement of tumor-associated macrophage infiltration, are useful biomarkers [23,28]. Recently, FAS ligands, one of the key components in cancer cell immunity, showed more immunoreactivity in WD-pNETs, a negative correlation with Ki-67, and lower expression in patients presumed to have a poorer prognosis [83]. In addition, nectin-3, a cell adhesion molecule that regulates the formation of tight junctions, was revealed as having an inverse association with tumor aggressiveness of pNENs [84]. Some studies have shown that low microvascular density (MVD) is an unfavorable prognostic factor [85,86,87]; however, other studies have argued that MVD is not a prognostic factor for survival [88,89]. According to a study that evaluated the role of tumor-associated angiogenesis as a prognostic factor, along with other methods in addition to MVD, the low solid area MVD, a high endothelial cell proliferation index, and high expression of CXCL-12 were associated with poor prognosis [87]. Interestingly, a recent study suggested that a novel, multiclassification, deep-learning pipeline can predict the risk of metastasis in pNENs patients, using hematoxylin and eosin sections of surgically resected tissue [90]. These results also suggest that prognostic morphological patterns exist among pNENs, both within the tumor and in the adjacent stromal regions.

**Table 3:** Clinicopathological prognostic factors for pNENs.

Factors	Prognosis	References
Clinical Factors		
Tumor, lymph node, metastasis (TNM) related factors	Disease status according to TNM stages associated with clinical outcomes	[7,9,22,23,24,25,26,27,28,29,30,31,32,33]
Age	Older age associated with poor OS	[24,26,32,40]
Functionality	Functional pNENs associated with better OS	[22,26,32]
Surgical resection	Surgical resection for pNENs showed better outcomes The absence of macroscopic radical surgery was associated with poor OS	[25,27,40,41]
^68^Ga-DOTATATE PET/CT	^68^Ga-DOTATOC PET/CT used to estimate the feasibility of peptide receptor radionuclide therapy The ^68^Ga-DOTANOC maximum standardized uptake values less than 37.8 are associated with disease progression ^68^Ga-DOTATATE total volume is associated with a decrement in progression-free survival (PFS) and increment in disease-specific mortality	[42,43,44,45]
Serum CgA	Serum CgA level has been associated with Ki-67 index, WHO classification, TNM stage, and treatment response Increased CgA levels are predictive of disease recurrence Early decrease in CgA after treatment is correlated with prolonged survival	[47,48,49,51]
Serum NSE	Serum NSE level is associated with overall survival (OS) and treatment response to everolimus	[47,53,54]
Serum pancreastatin	Higher pancreastatin levels are significantly associated with worse PFS and OS Negative pancreastatin response to somatostatin analogs has been associated with poor OS	[57,58]
Albumin-to-alkaline phosphatase ratio	Higher serum albumin-to-alkaline phosphatase ratio have shown more favorable prognosis	[59]
Pancreatic duct dilatation and neutrophil-to-lymphocyte ratio	An independent predictor of OS and disease-free survival for pNENs of the pancreatic head	[60]
**Pathological Factors**		
WHO classification/Ki-67/mitotic rate/grade/differentiation	WHO classification presents excellent criteria regarding the overall prognosis of pNENs The reported 5-year survival rates, according to the WHO classification, are as follows: G1 is 75%, G2 is 62%, and G3 is 7% High cell proliferation is associated with poor clinical outcomes	[3,10,21,22,23,25,27,28,29,30,31,32,33,41,69,70,71,72,76]
Lymph node ratio	A strong prognostic factor for DFS and OS in resected PNETs Lymph node ratio ≥ 0.5 is independently associated with worse disease-specific survival	[72]
Neuroinvasion or vascular invasion	Neuroinvasion or vascular invasion is associated with poor outcomes	[33]
Peritumoral or stromal infiltrative patterns	The type of growth of pNENs with peritumoral infiltrating and stromal desmoplastic reaction has shown poor clinical outcomes	[33]
Tumor-associated macrophage infiltration, high CD68 score	A useful biomarker to predict recurrence in surgically resected nonfunctional pNENs	[23,28]
FAS ligand	FAS ligands have shown more immunoreactivity in WD-pNENs, a negative correlation with Ki-67, and less expression in patients presumed to have poorer prognosis	[83]
Nectin-3	Decreased nectin-3 expression in the membrane is associated with increased tumor aggressiveness of pNENs	[84]

##### Risk Stratification Model with Clinicopathological Factors

Attempts to predict the prognosis of pNENs using a combination of various clinicopathological factors have been made due to the limited predictive power of each single prognostic factor [22,30,31,32,91]. TNM staging systems, including AJCC or ENETS, showed fine predictive efficacy, especially in comparing pNENs with or without distant metastasis. However, the prediction of recurrence or survival in resected pNENs has a different goal, which is the selection of optimal patients for adjuvant therapy in nonmetastatic pNENs; in addition, predicting survival in resected pNENs with distant metastasis is challenging.

A combination scoring system of tumor grade, positive lymph nodes, and perineural invasion for surgically resected grade 1 or 2 nonfunctional pNENs is an independent predictor for tumor recurrence [91]. Patients with a recurrence score of more than 24 are considered high risk for disease recurrence and may benefit from adjuvant therapy. Another study on functional and nonfunctional WD-pNETs from grades 1 to 3 with R0 resection reported that a simplified risk stratification, in combination with functionality, Ki-67 index, and a ENETS TNM-staging-based T/N stage, discriminated patients based on DFS [22]. A simple prognostic model based on Ki-67 index, age, and sex showed good efficacy in predicting the survival of surgically resected nonfunctional pNENs with or without distant metastasis [31]. The prognostic score with age, differentiation, and distant metastasis correlated with outcomes and offered excellent survival discrimination [32]. The prognosis of pNENs with synchronous liver metastasis was evaluated in combination with the distribution of liver metastasis, the size of the metastatic mass, tumor grade, and surgical resection, regardless of its purpose [30]. Although no robust prediction system for multiclinicopathologic factors has been presented yet, the utilization of molecular-level risk factors introduced in this article may improve the accuracy of future prediction system.

#### 2.2.2. Molecular Prognostic Factors

The exploration of de novo molecular-level prognostic factors is both important and challenging. Although various prognostic factors have been proposed, a single omnipotent integrated prediction method has not yet been presented. Research on pNENs pathophysiology and molecular-level prognosis prediction has made tremendous progress. A highly valuable approach for the differentiation of subtypes, tumor heterogeneity, and unexplainable features of pNENs by clinicopathological features is through a molecular-level evaluation of pNENs. Recently, a substantial number of novel biomarkers with better efficacy for prognostic prediction of pNENs have been introduced, and the molecular prognostic factors stratified in three categories, including the latest findings, are described as follows: genetic factors, epigenetic factors, and tumor microenvironments.

##### Genetic Prognostic Factors for pNENs

1) Inherited Syndromes

In the genetic evaluation of pNENs, it is important to first assess the inherited syndromes, which determine patients at risk for the development of pNENs through germline mutations (Table 4). Patients who have germline mutations in multiple endocrine neoplasia 1 (MEN1), von Hippel-Lindau syndrome (vHL), neurofibromatosis (NF1), and tuberous sclerosis (TSC complex) may have pNENs at diagnosis or during follow-up [92]. The percentage of pNENs with inherited syndromes is relatively small, with approximately 5–10% in overall pNENs [69]. pNENs in these patients have limited impact on OS and have slow-growing characteristics in comparison to sporadic pNENs [69].

2) Sporadic pNENs

The most common genetic mutations in sporadic pNENs are MEN1, death domain-associated protein (DAXX), alpha thalassemia/mental retardation X-linked chromatin remodeler (ATRX), CDKN2A, and AKT/mammalian target of rapamycin (mTOR) pathway-related genes, such as mTOR, PIK3CA, AKT1, PTEN, tuberous sclerosis complex 1 (TS1), tuberous sclerosis complex 2 (TSC2), and ataxia telangiectasia mutated (ATM) (Table 4).

3) MEN1

Menin is a histone methyltransferase complex that is encoded in MEN1, a well-known tumor-suppressor gene. MEN1 mutation is detected in 28–44% of sporadic pNENs and is associated with better prognosis [16,93,94,95].

4) DAXX/ATRX

DAXX/ATRX constitutes a transcription and chromatin-remodeling complex, and DAXX/ATRX mutation is detected in 28–43% of sporadic pNENs and is associated with alternative lengthening of telomeres (ALT) [4,16,96]. The predictive role of DAXX/ATRX has shown inconsistency in previous studies. Some studies have reported that DAXX/ATRX mutation is correlated with tumor stage and metastasis, and is associated with aggressive clinical behavior, reduced recurrence free survival (RFS) time, and worsening of the tumor-associated survival period [4,97,98,99]. However, some other studies have reported that DAXX/ATRX mutation is associated with better prognosis and prolonged OS in pNENs with distant metastasis [16,100]. Interestingly, according to the disease status of pNENs and mutational status of DAXX/ATRX genes, significantly different and complex survival outcomes have been observed, and DAXX/ATRX protein expression was an independent prognostic factor associated with poor OS and poor survival after recurrence. However, DAXX/ATRX protein expression seemed to be associated with a longer DFS for curative resection of pNENs [95]. Recently, ATRX/DAXX loss and ALT have been associated with several adverse prognostic findings and distant metastasis/recurrence [101].

5) Genetic Alteration in AKT/mTOR Pathway

The growth and apoptosis of pancreatic β cells are regulated by the mTOR pathway [102]. Key components of the mTOR pathway, including TSC2 or PTEN, are downregulated in 80% of pNENs [94,103]. Gene mutations involved in the mTOR pathway are detected in 14–38% of sporadic pNENs, and are more frequently mutated in Asians [16]. Overexpression of the components of the mTOR pathway is associated with higher proliferation, distant metastasis, and poor disease-specific survival [104]. Loss of TSC2 or PTEN has been associated with shorter OS, and a loss of TSC2 or ATRX has been associated with shorter RFS. Loss of ATRX or TSC2 has been significantly associated with lymph node metastasis, and combined loss of TSC2 and ATRX has been determined as an independent prognostic factor for shorter RFS in G2 pNENs [105].

6) Genetic Alteration in Angiogenesis Pathway

Gene mutations related to angiogenesis, such as VHL, ANGPT1, ANGPT2, and HIF1A, were detected in 45% of sporadic pNENs, and these mutations were more frequently mutated in Asians [94]. Moreover, G1 tumors had a higher incidence of mutations in the angiogenesis-pathway genes than in G2 and G3 tumors.

7) Miscellaneous

Loss of CDKN2A was detected in 30% of pNENs and it has been associated with distant metastasis and poor survival [106]. Somatostatin receptor 2A (SSTR2A) is usually more highly expressed in WD-pNETs [107]. A lower Ki-67 index significantly correlated with SSTR2A expression, and a strongly positive SSTR2A was associated with longer survival [108]. Loss of O-6-methylguanine-DNA methyltransferase (MGMT) is associated with radiological objective response, better PFS, and a more favorable response to alkylating agents [109,110,111,112]. ARID1A is a tumor-suppressor gene and a large subunit of the switch/sucrose nonfermentable (SWI/SNF) complex [113]. ARID1A mutation with reduced expression has been related to poor prognosis, which is associated with a higher Ki-67 index, aggressive clinicopathological features, and liver metastasis [114].

**Table 4:** Genetic prognostic factors for pNENs.

Factors	Efficacy/Details	References
Germline mutations in inherited syndromes	Small percentage of pNENs (10%) Multiple endocrine neoplasia 1 (MEN1), von Hippel-Lindau syndrome (vHL), neurofibromatosis (NF1), tuberous sclerosis (TSC complex) Slow-growing pNENs Limited impact on overall survival	[69,92]
MEN1	Detected in 28–44% of sporadic pNENs Associated with better prognosis	[16,93,94,95]
DAXX/ATRX	DAXX and ATRX chromatin remodeler (ATRX) Detected in 28–43% of sporadic pNENs Associated with alternative lengthening of telomeres (ALT) ATRX/DAXX loss and ALT were associated with several adverse prognostic findings and distant metastasis/recurrence Conflicting predictive value ✓ Aggressive clinical behavior, reduced DFS, and OS ✓ Better prognosis, prolonged OS in pNENs with distant metastasis	[4,16,94,95,96,97,98,99,100,101]
Akt/mTOR pathway	Mammalian target of rapamycin (mTOR) regulates cell proliferation mTOR/PIK3CA/AKT1/PTEN/TS1/TSC2/ATM mTOR downregulates TSC2 and phosphatase and tensin homolog (PTEN) Detected in 14–38% of sporadic pNENs More frequently mutated in Asians Overexpression of mTOR or its downstream targets is associated with higher proliferative capacity and poorer prognosis Loss of PTEN or TSC2 is correlated with advanced-stage disease, a higher Ki-67 index, and shorter DFS and OS	[16,94,102,103,104,105]
Angiogenesis pathway	VHL/ANGPT1/ANGPT2 /HIF1A Detected in 45% of sporadic pNENs More frequently mutated in Asians	[94]
CDKN2A	CDKN2A Detected in 30% of sporadic pNENs Associated with metastasis and shorter survival	[106]
SSTR2A	Somatostatin receptor 2A (SSTR2A) More highly expressed in WD-pNETs Assaying SSTR2A expression by immunohistochemistry provided no additional value compared to assaying SRS uptake in predicting response to peptide receptor radionuclide therapy	[107,108]
MGMT	Methylguanine-DNA methyltransferase (MGMT) Loss of MGMT expression is associated with increased PFS and a more favorable response to alkylating agents	[109,110,111,112]
ARID1A	ARID1A (AT-rich interactive domain-containing protein 1A, BAF250A) is a large subunit of the SWI/SNF complex The expression of ARID1A was remarkably downregulated in nonfunctional pNENs and corresponding liver metastases Reduced expression of ARID1A was associated with malignant clinicopathological features The loss of ARID1A was related to a high Ki-67 index Patients with ARID1A-negative expression had a significantly worse OS rate than those with ARID1A-positive expression	[114]

##### Epigenetic Prognostic Factors for pNENs

As a result of the genetic landscape of pNENs by next-generation sequencing and other high-throughput advanced molecular techniques, recently, a surge of novel information on epigenetic alterations accompanied by genetic changes has surfaced. Due to the paucity of genetic mutations in common tumor-suppressor genes and oncogenes, attention has shifted to epigenetic alterations in the oncogenic pathway and tumor evolution in pNENs. Beyond genetic alterations, it is well known that epigenetic changes have been observed in pNENs and the adjacent normal parenchyma, which seem to be involved in the pathophysiology of pNENs [24]. In this section, a comprehensive review of epigenetic prognostic factors for pNENs was fulfilled (Table 5).

1) DNA Methylation

The patterns of DNA methylation were associated with clinical and genomic information, and they were divided into three subgroups: T1, T2, and T3 [115]. DNA methylation affects carcinogenesis and prognosis in pNENs. The T1 subgroup included tumors with heterogeneous patterns of copy number alterations, and this subgroup was enriched for functional tumors, as well as DAXX/ATRX and MEN1 wild-type genotypes. Tumors in the T2 subgroup contained mutations in DAXX/ATRX and MEN1 with recurrent patterns of chromosomal losses in half of the genome, which have been associated with a reduced survival in pNENs. Tumors with the T2 subgroup pattern methylation were larger and harbored more somatic point mutations than the other two subgroups. The T3 subgroup contains mutations in MEN1 with a recurrent loss of chromosome 11. T3 tumors had less aggressive behavior and a higher proportion of G1 tumors compared with other groups. Furthermore, more accurate tumor-type identification is possible with the analysis of methylation profiling in comparison with genomic mutations, copy number alterations, or immunohistochemistry of single-cell-type markers, such as PDX1, ARX, and SOX9, in further characterizing pNECs [116]. In this study, a robust and clinically applicable method to clearly distinguish pNECs from G3 pNENs was introduced based on methylation profiling to improve patient stratification.

2) DAXX/ATRX

DAXX/ATRX proteins are part of chromatin-modifying complexes affecting histone and chromatin modification, and histone H3.3 chaperone, which is guided by ATRX, is a nuclear protein of the SWI/SNF complex of chromatin-remodeling genes [16,117]. Accordingly, alteration in the DAXX/ATRX gene plays an important role in epigenetic modification. As ATRX recruits DAXX, DAXX/ATRX deposits H3.3 at the H3K9 me3-enriched chromatin and telomeres to modify chromatin remodeling and telomere lengthening. The loss of DAXX/ATRX leads to aggravation in DNA damage, worsening genomic instability, and alterations in the ALT pathway [96,98]. ATRX/DAXX loss/mutations and ALT positivity are associated with a more aggressive phenotype, such as a larger tumor size, high grade, advanced stage, chromosomal instability, metastatic disease, and poor survival [16,96,98,101,117].

3) Aristaless-Related Homeobox (ARX) Gene and Pancreatic and Duodenal Homeobox 1 (PDX1)

Recently, whole transcriptome and epigenome studies have revealed the differential expression of ARX and PDX1, which are known as transcription factors that may determine the risk of metastatic disease [118,119]. PDX1 expression is associated with indolent features and ARX expression is associated with aggressive features of pNENs. Furthermore, the expression of these transcription factors in pNENs has recently been reported to be prognostic factors for RFS [118].

4) Ras-Association Domain Family 1 (RASSF1) Gene

RASSF1 is a tumor-suppressor gene that causes G1 cell cycle arrest through the accumulation of cyclin D1 [120]. This pathway has been downregulated in pNENs universally secondary to promoter-selective methylation that leads to the production of only RASSF1A and RASSF1C [120,121,122,123]. RASSF1A regulates cellular proliferation, apoptosis, and the stabilization of microtubules, and promoter methylation of RASSF1A was associated with large tumors greater than 5 cm, lymph node involvement, and hepatic metastasis [121,122,123,124,125]. Promoter methylation of RASSF1A was not detected in normal tissue adjacent to pNENs, which suggested that RASSF1A may have a role in the development of pNENs. RASSF1C inhibits β-catenin degradation, and, as a result, the Wnt pathway was upregulated, which suggested that RASSF1C may have a role in the development of pNENs [126,127]. Overexpression of RASSF1C has not been associated with promoter methylation; however, RASSF1C is released on DAXX degradation with DNA damage.

5) CDKN2A

CDKN2A encodes a tumor-suppressor protein, p16, that regulates entry into the S-phase of the cell cycle. CDKN2A hypermethylation, seen in 40% of pNENs, is associated with distant metastasis and poor PFS, which is also an independent negative predictor of post-surgical survival [122,124,125,128].

6) Tissue Inhibitor of Metalloproteinase-3 (TIMP-3)

TIMP3 is a tumor-suppressor gene that inhibits metalloproteinase, and it reduces cellular growth, cellular migration, and invasion. Approximately 44% of pNENs had TIMP-3 promoter hypermethylation, and these alterations were more frequently found in pNENs with more aggressive clinical behavior, including distant metastasis [129,130].

7) MGMT

To prevent DNA cross-links, MGMT is a DNA repair enzyme that allows rapid reversal of alkylation of the O-6 position of guanine bases prohibiting the repair process [131]. Hypermethylated promoter of MGMT has been observed in 17–50% of pNENs [122,132]. This feature was associated with increased PFS and a more favorable response to alkylating agents as treatment for well-differentiated advanced NETs. However, studies have presented conflicting results between MGMT expression and response to temozolomide in pNENs [110,111,131,133,134,135]. Additionally, the relationship between methylation of the MGMT gene body and its expression remains unclear [115]. To evaluate the value of tumor MGMT promoter methylation in the prediction of the objective response in patients treated with temozolomide and streptozotocin, a clinical trial has recently been conducted [136]. Moreover, the value of MGMT immunohistochemistry and the efficacy of temozolomide and streptozotocin treatment in comparison with oxaliplatin-based chemotherapy were also evaluated (NCT03217097). To confirm the true therapeutic prediction efficacy and prognostic prediction of MGMT, it is noteworthy to focus on the results of this study in the future.

8) Insulin Growth Factor-2 Gene (IGF2)

Described in the majority of insulinomas and subsets of other pNENs, the loss of imprinting and overexpression from the IGF2 gene locus are a renowned epigenetic event [137,138,139]. A decreasing degree of methylation in the different IGF2 regions correlated with increasing degree of malignancy [139].

9) Promoter Methylation of MutL Homolog 1 (MLH1)

MLH1 is one of the mismatch repair genes. The association between aberrant promoter methylation MLH1 and microsatellite instability (MSI) in pNENs has been proven, and MSI-high pNENs are often associated with a favorable prognosis [122,140]. Reduced expression of the MHL1 protein was detected in 36% and showed correlation with high MSI [141]. Detection of these two markers showed a significant association with tumor malignancy and incurable disease.

10) CIMP

CIMP is defined as a simultaneous hypermethylation of numerous CpG islands surrounding the promoter regions of several genes to inhibit physical binding of transcription factors [142]. CIMP frequently occurs in 83% of pNENs. It has been associated with a high Ki-67 index proliferation of over 10%, and it also showed correlation with distant metastasis and poor survival outcomes [132].

11) MEN1

MEN1 is part of chromatin-modifying complexes regulating histone and chromatin modification to maintain transcription at multiple loci-encoding cell cycle regulators essential for endocrine growth control [143]. Menin affects histone H3 methylation status and recruits the nuclear complex mixed-lineage leukemia 1 and 2 (MLL1 and MLL2), which binds to promoter regions of CDK inhibitors (CDKN2C and CDKN1B) to inhibit tumor formation [144,145]. Menin inhibition increases glucagon-like-peptide-1 (GLP-1) receptor levels, GLP-1 agonist-mediated phosphorylation of FOXO1 and CREB, and cell proliferation in islets [146]. Menin may function as a tumor suppressor by regulating histone methylation states of the specific target gene promoters that control proliferation and tumorigenesis [147]. Chromatin modification has multiple interacting counterparts, and menin might act as a transcriptional activator through the MLL complex or as a repressor through HDAC/SUV39H1 in different target genes, which provide an efficient epigenetic regulatory mechanism for cell cycle and cell tumorigenesis. Depending on the gene contexts, these two types of histone modifications could occur in an independent or a synergistic manner.

12) Long Interspersed Nucleotide Element 1 (LINE1) and Arthrobacter Luteus (ALU) Homolog

LINE1 and ALU homolog are noncoding genomic repetitive sequences, and the methylation status of these lesions has been interpreted as a surrogate marker of global hypomethylation, which is generally considered a poor prognostic marker in most solid tumors [148,149]. Hypomethylation of LINE1 was identified in 100% of pNENs, and hypomethylation of LINE1 and ALU homolog is associated with advanced stages and poor prognosis [130,149,150].

13) MicroRNA (miRNA)

MicroRNAs are small (20 to 24 nucleotides) molecules from noncoding RNA gene products and are extensively involved in gene regulation, such as cell proliferation and apoptosis, by negatively regulating their target messenger RNAs [151]. The increased expression of miR-103 and miRNA-107 associated with a reduced expression of miRNA-155 discriminates tumors from normal; miR-204 expression primarily occurs in insulinomas and correlates with immunohistochemical expression of insulin. miRNA-21 overexpression is strongly associated with Ki-67 proliferation index, hepatic metastasis, and survival, according to an extensive survey study of miRNA expression in normal pancreas, pNENs, and acinar carcinoma [152,153]. The other global miRNA profiling of pNENs reported that the expression of miRNA-642 correlated with Ki-67 and the expression of miRNA-210 correlated with metastatic disease, but not miRNA-21 [154]. The miRNA-196a level was significantly associated with stage and mitotic count, and high miRNA-196a expression was significantly associated with decreased OS and DFS with high risk for recurrence in resected pNENs [155]. Moreover, overexpression of miRNA-3653 may be associated with an increased risk of metastatic disease in pNENs, probably through the ATRX and ALT pathway interaction [156]. The miR-96-5p expression level was increased along with tumor grade, and its target FoxO1 expression decreased along with tumor grade [157].

To manage pNENs, it is important to evaluate and subtype according to the comprehensive expression of miRNAs. Recently, a large study with an extensive exploration of pNENs using dual miRNA and mRNA transcriptome profiling analysis revealed that the subtypes of three distinctive differential expression of 30 miRNAs were identified as miR-cluster-1 (well-differentiated islet/insulinoma tumors), miR-cluster-2 (poorly differentiated tumors associated with liver metastases), and miR-cluster-3 (dubbed metastasis-like primary and specific gene mutation-enriched subtype) [158].

**Table 5:** Epigenetic prognostic factors for pNENs.

Factors	Efficacy/Details	References
DNA methylation pattern	Three subgroups of pNENs, termed T1, T2, and T3, with distinct patterns of methylation ▪ The T1 subgroup was enriched for functional tumors and ATRX, DAXX, and MEN1 wild-type genotypes ▪ The T2 subgroup contained tumors with mutations in ATRX, DAXX, and MEN1 and recurrent patterns of chromosomal losses in half of the genome with no association between regions with recurrent loss and methylation levels. T2 tumors were larger and had lower methylation in the MGMT gene body, which showed positive correlations with gene expression ▪ The T3 subgroup harbored mutations in MEN1 with recurrent loss of chromosome 11, was enriched for grade G1 tumors, and showed histological parameters associated with better prognosis Methylation plays a role in driving tumorigenesis and potentially stratifying prognosis in pNENs Methylation profiling is a superior method of tumor-type identification to genomic mutations, copy number alterations, or IHC of single markers Distinguish pNECs from G3 pNETs, improving patient stratification	[115,116]
DAXX/ATRX	Part of chromatin-modifying complexes affecting histone and chromatin modification Histone H3.3 chaperone which is guided by ATRX and is a nuclear protein of the SWI/SNF complex of chromatin-remodeling genes ATRX recruits DAXX, mediating DAXX-dependent H3.3 deposition at H3K9 me3-enriched chromatin and telomeres where it mediates both chromatin remodeling and telomere length Loss-of-function mutations in DAXX and ATRX lead to an exaggerated DNA damage response, ALT pathway, and genomic instability Previous studies have illustrated 100% concordance with DAXX or ATRX mutations and the ALT phenotype in pNETs ATRX/DAXX loss/mutations and ALT positivity are associated with a more aggressive tumor phenotype (larger tumors, grade, and stage), chromosomal instability, metastatic disease, and survival, with the absence of ATRX/DAXX being an independent predictor of survival in multivariable analysis	[16,96,98,101,117]
Aristaless-related homeobox gene (ARX), pancreatic and duodenal homeobox1 (PDX1)	Additionally, whole transcriptome and epigenome studies have found the differential expression of transcription factors, and ARX and PDX1 can also determine the risk of metastatic disease. PDX1 expression is typically associated with an indolent clinical behavior, while the expression of ARX or the lack of both proteins correlates with an aggressive disease course. These transcription factors in pNENs were recently reported to be a prognostic biomarker for RFS, independent of the tumor size, WHO grade, and ALT	[118,119]
Ras-association domain gene family 1 (RASSF1)	Promoter hypermethylation in pNENs A tumor-suppressor gene which functions to arrest the cell cycle in G1 through a mechanism that leads to the accumulation of cyclin D1 Downregulation of this pathway is almost universally secondary to promoter methylation Selective methylation leads to the production of RASSF1A and RASSF1C only RASSF1A ▪ Regulates cellular proliferation, apoptosis, and stabilization of microtubules ▪ Association between a higher frequency of promoter methylation with tumors larger than 5 cm and those with either lymph node or hepatic metastases RASSF1C ▪ RASSF1C plays a role in the upregulation of the Wnt pathway through the inhibition of theβ–catenin degradation ▪ Death domain-associated protein (DAXX) retains RASSF1C within the nucleus, releasing RASSF1C on DAXX degradation with DNA damage	[109,110,120,121,122,123,124,125,126,127,132]
Cyclin-dependent kinase inhibitor 2a/P16INK4a (CDKN2A)	Encodes the tumor-suppressor protein p16 Primary role is to regulate the S-phase of the cell cycle To be silenced in many tumor types through promoter methylation of the CDKN2A/P16 locus CDKN2A methylation is present in 40–57% of pNENs Hypermethylation of CDKN2A may be an independent negative predictor of patient survival following surgical resection, a feature that has been shown to be associated with metastases and poor 5-year PFS	[122,124,125,128]
Tissue inhibitor of metalloproteinase-3 (TIMP3)	Tumor-suppressor gene inhibits metalloproteinase Reduces cellular growth, cellular migration, and invasio A total of 44% of pNENs revealed TIMP-3 alterations with promoter hypermethylation TIMP-3 alterations are more frequently found in pNENs with metastasis	[129,130]
O-6-alkylguanine-DNA alkyltransferase (MGMT)	MGMT promoter methylation is associated with increased PFS and a more favorable response to alkylating agents MGMT inhibits the binding of transcription factors and other cellular regulators to gene promoters MGMT hypermethylation was observed in 17–50% of pNENs A significantly longer median PFS was observed in patients with MGMT promoter methylation treated with an alkylating agent for well-differentiated, advanced pNENs	[110,111,115,122,131,132,133,134,135,136]
Insulin growth factor-2 gene (IGF2)	A gene that is imprinted through hypermethylation Loss of imprinting and overexpression of IGF2 have been described in pNENs Hypermethylation of region two was specific to insulinomas Increasing degree of malignancy and a decreasing degree of methylation in IGF2	[137,138,139]
Promoter methylation of MLH1	Associated with microsatellite instability Presence of promoter methylation of MLH1 and microsatellite instability was associated with poor prognosis	[122,140,141]
CpG island methylator phenotype (CIMP)	Observed in 83% of pNENs Associated with metastases and worse prognosis	[132]
MEN1	Part of chromatin-modifying complexes affecting histone and chromatin modification Menin regulates the methylation of histone H3 at lysine residue 4 (H3K4 me3) and recruits the nuclear complex mixed-lineage leukemia 1 and 2 (MLL1/2) MLL binds to the promoter regions of cyclin-dependent kinase inhibitors (CDKis) p18Ink4c (CDKN2C) and p27Kip1 (CDKN1B), maintaining the expression of these genes and inhibiting tumor formation With MLL loss or with disruption of this complex by menin, methylation levels are reduced, resulting in reduced CDKi expression and tumor growth MEN1 has also been observed to interact with histone deacetylase (HDAC) and histone methyltransferases, including SUV39H1, acting as either an activator or suppressor of gene transcriptional activity	[143,144,145,146,147]
Long interspersed nucleotide element 1 (LINE1) and Arthrobacter luteus (ALU) homolog	Noncoding genomic repetitive sequences Used as a surrogate marker of global hypomethylation Hypomethylation of these regions is associated with poor prognosis	[130,149,150]
MicroRNA (miRNA)	miRNAs are one of the most abundant classes of gene-regulatory molecules Increased expression of miRNA-103 and miRNA-107 and reduced expression of miRNA-155 in tumor tissue compared to normal pancreatic tissue miRNA-21 overexpression in pNENs ▪ Strongly associated with both a high Ki-67 proliferation index and the presence of liver metastases ▪ Associated with Ki67 and the presence of metastatic disease and survival Expression of miRNA-642 correlated with Ki67 Expression of miRNA-210 correlated with metastatic disease miR-196 upregulation and high expression correlated with aggressive behavior, poor prognosis, and decreased disease-free and OS High expression of miR-3653 and low expression of miR-4417, miR-574-3p, and miR-664b-3p are associated with distant metastasis miR-96-5p levels increased with tumor grade Three distinct subsets which differentially expressed 30 distinct miRNAs ▪ miR-cluster-1 included MEN1 mutant tumors with moderate metastatic potential ▪ miR-cluster-2 was enriched in metastasis-like primaries (MLP) with high metastatic potential ▪ miR-cluster-3 predominantly included insulinomas, none of which were associated with metastatic disease	[152,153,154,155,156,157,158]

#### 2.2.3. Recent Advances in Multifactorial Prediction Models, including Molecular Biomarkers

The development of next-generation sequencing techniques has allowed the genetic mapping of pNENs, and molecular subtyping in pNENs includes classifications based on common multigene mutations, a large-scale loss of heterozygosity or copy number variations, and an islet cell type-specific signature. Moreover, molecular subtyping provides insights into solving the unmet needs from clinicopathological prediction [4,159,160,161,162,163]. However, despite the advances in multiomics sequencing, including high-throughput transcriptomics and epigenetic sequencing, an in-depth understanding of pNENs remains insufficient [164]. The application of molecular profiling of pNENs in real practice to determine treatment is challenging because a few detected targetable alterations have been associated with treatment options outside of permission [165]. Still, molecular biological profiles can be used in terms of the classification of pNENs subtypes and prognosis predictions. At least 20% of the tumor portion from the tumor tissue is needed to identify the molecular profile of pNENs; however, the remnant or archived tissue after standard diagnostic process is usually small and the quality is unguaranteed for a reliable molecular analysis. In this context, circulating tumor DNA (ctDNA) has been proposed as one of the de novo alternatives in pNENs, which can be easily obtained, and it can provide information from fresh viable tissue [166,167]. Additionally, exploration with ctDNA is possible to track changes in tumor burden or molecular profile according to time changes or according to treatment, and it is one of the several attractive points of ctDNA.

##### Classification of pNENs based on Multiomics Sequencing

The classification of pNENs with cell origin has gained more attention due to recent multiomics development. The simple pNENs subtypes have been divided into α-cell-like and β-cell-like pNENs. Furthermore, the somatic mutations of MEN1 and DAXX/ATRX exclusively occur in α-cell-like pNENs. The suggested classification based on multiomics features enables a more detailed classification of pNENs to achieve an in-depth understanding of the disease and to predict clinical outcomes more precisely. However, quite a few differences exist in the involved molecular factors between each classification; thus, a more integrated classification method is needed in the future.

##### Classification of the Cell Origin of pNENs: α-Cell-Like or β-Cell-Like Tumors

pNENs have been subdivided based on ATRX, DAXX, and MEN1 (A-D-M) mutations, and this was correlated with a worse prognosis than tumors with wild-type A-D-M mutation [119]. A-D-M-mutated pNENs showed high ARX and low PDX1 gene expression with PDX1 promoter hypermethylation, and they possessed a gene expression signature related to that of the α-cells of the pancreatic islets, including increased HNF1A and transcriptional target gene expression. In this study, ARX and IRX2 gene expression is specific for α-cell-like pNENs and PDX1 gene expression is specific for β-cell-like pNENs.

Another study suggested the classification of pNENs according to DNA methylation profiles of IRX2, ARX, and PDX1 that were divided into α-cell-like and β-cell-like subtypes [168]. They were further classified into different subtypes based on mutations of MEN1 and DAXX/ATRX and CNV, while the mTOR and Hippo pathways were enriched in α-cell-like tumors.

Furthermore, another study suggested nonfunctional pNENs subtypes based on the enhancer signature, such as α-cell-like or β-cell-like and intermediate tumors [118]. In this study, distant relapses predominated in patients with α-cell-like (ARX+) tumors.

Another study showed different classifications according to the DNA methylation patterns to DNA mutation patterns, and nonfunctional pNENs were divided into three subtypes as follows: α-cell-like, β-cell-like, and intermediate pNENs [169]. Intermediate tumors have a higher risk of relapse compared to α- and β-like tumors, and they harbor frequent MEN1 and DAXX/ATRX mutations and whole chromosome loss. Depending on DNA methylation similarity to α- or β-cells, pNENs have different mutational profiles, disease stages, and prognosis.

##### Multiomics Profile and Histology Classification

Sporadic pNENs are clustered into three subtypes as islet/insulinoma (IT), metastasis-like primary (MLP), and intermediate pNENs according to a comprehensive multiomics profile and histology [158]. MLP-subtype pNENs express pancreatic progenitor or immature genes with higher frequency of MEN1 and DAXX/ATRX gene mutations than others. The IT subtype shows features of mature β-cells with INS and PDX1 gene expression, and intermediate pNENs show mature β-cell features with expression of GCG, NKX2-2, and GATA6 genes. These findings suggest different tumorigenesis pathways, and these subtypes exhibit distinct metabolic profiles marked by differential pyruvate metabolism, substantiating the significance of their separate identities.

Recently, another proteotranscriptomic classification and characterization of pNENs were introduced [170]. In this study, metabolism-related molecular differences in an α-cell-like subgroup and the involvement of the Hippo signaling pathway in a stromal/mesenchymal subgroup were uncovered, and pNENs were subclassified into four subgroups as α-cell-like, stromal/mesenchymal, proliferative, and PDX1-high types. Mutant MEN1/DAXX and metabolic features characterize an α-cell-like subgroup. The stromal/mesenchymal subgroup has elevated YAP1 and WWTR1 activities. The proliferative subgroup exhibiting molecular features indicative of increased cell proliferation consisted of roughly equal proportions of pNETs and pNECs, which suggested that a subset of pNETs is more similar to pNECs than other pNETs at the molecular level.

##### Advances in Liquid Biopsy for pNENs

The need for sufficient tumor tissue to pass the quality examination to perform molecular analysis and the associated adverse events of tissue biopsy are major limitations of using molecular biomarkers on tissue. Moreover, the measurement of changes in conditions over time or treatment in tissue-based molecular evaluation is difficult. Liquid biopsy could be an alternative to tissue biopsy and blood is currently the most studied liquid biopsy, including several molecules that can be analyzed to evaluate pNENs.

Epithelial cell adhesion molecules were expressed in circulating tumor cells (CTCs) of pNENs [171]. The presence of CTCs has been associated with progressive disease, bone metastasis, PFS, and OS [172,173,174]. The number of CTCs has potential as a surrogate marker for prognosis and treatment response, because it has been associated with tumor burden, and changes in CTCs demonstrate the association between treatment response and OS [175]. Additionally, in the phase II PAZONET study, CTCs are one of the potential biomarkers for selecting patients for pazopanib [176]. It seems that the use of CTC in the evaluation has great potential for development, because the analysis of expression of molecular markers and genetic and epigenetic alterations have been detected in isolated CTCs beyond the detected number of CTCs [174,177,178,179].

Recently, the presence of tumor-specific genetic alterations in ctDNA from plasma of metastatic pNENs was detected with droplet digital polymerase chain reaction, and its concordance with fresh frozen tumor tissue and buffy coat was confirmed [180]. In this study, CNVs could be detected in ctDNA with shallow whole-genome sequencing, and it showed potential as a prognostic factor, which showed an increase in ctDNA concentration, ctDNA level, and chromosomal aberrations in parallel with disease progression. DNA methylation patterns could be detected in ctDNA, and the recent MGMT-NET trial was initiated to confirm the value of detectable MGMT hypermethylation in ctDNA [136]. Several marked DNA methylation patterns have been proposed in various studies; however, these markers are not yet actively evaluated in ctDNA.

The RNA extracted from whole-blood samples of pNENs patients and candidate biomarkers were evaluated and the NETest algorithm was developed [181,182]. NETest consists of a PCR-based 51-transcript signature, and it significantly outperformed chromogranin A, neurokinin A, pancreastatin, and single analyte tests [183,184]. This test has proven its clinical utility in a large prospective comparative cohort study including 359 pNENs (65% of metastatic pNENs); it was concordant with imaging, accurately classified progressive disease, and predicted tumor recurrence after surgery [185]. Furthermore, the dynamics in the NETest score were ancillary to obtain a clue to early objective genomic identification of residual disease, while R0 resection appears to be ineffective in approximately 30% of patients [186].

The use of molecular profiling utilizing liquid biopsy samples in pNENs is feasible and various studies have reported promising results. It will be an essential factor in diagnosis and prediction of prognosis in the near future, with advances in analyses of combinatorial mutational profiles, epigenetic modifications, and further discovery of de novo biomarkers using liquid biopsy.

## 3. Conclusions

**Figure 1:**
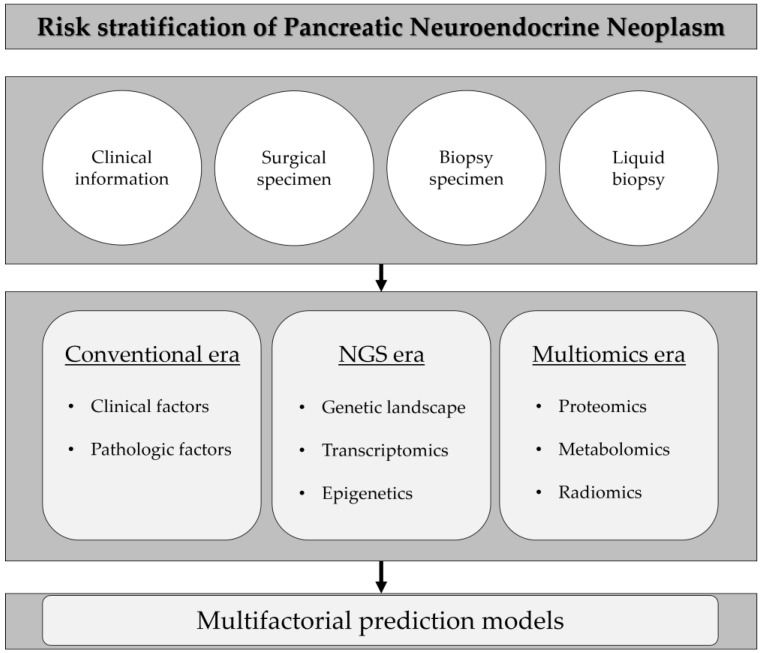
A schematic diagram for risk stratification of pancreatic neuroendocrine neonplasm.

pNENs consist of heterogeneous tumors, and risk stratification for predicting the exact prognosis of the disease still remains challenging. Sophisticated classifications and classical, as well as novel, biomarkers were thoroughly reviewed in this article. To predict prognosis and provide optimal management, it is very important to refine the clinical, pathological, molecular, and epigenetic properties of pNENs (Figure 1). Owing to advances in sequencing technology, the genetic landscape of pNENs has been revealed and knowledge of the molecular features of pNENs has been systematically advanced. In the future, multiomics analysis including high-throughput transcriptomics, epigenomic analysis, proteomic analysis, metabolomics analysis, and radiomic analysis will determine more in-depth prognostic factors, especially for the roles of genetic mutations, non-coding RNAs, epigenetic signatures, metabolites, and radiologic features in pNENs. In addition, prognostic prediction with a combination of various factors, rather than a single factor, seems to be more efficacious, and prognostic factors through noninvasive methods are expected to develop the further advancement of liquid biopsy in the future.

## Figures and Tables

**Table 1:** World Health Organization classification of pNEN.

	pNET Grade 1		pNET Grade 2		pNET Grade 3		pNEC Grade 3	
	Mitoses/10 HPF	Ki-67 Index	Mitoses/10 HPF	Ki-67 Index	Mitoses/10 HPF	Ki-67 Index	Mitoses/10 HPF	Ki-67 Index
**2010**	<2	<3%	2–20	3–20%	NA	NA	>20	>20%
**2017**	<2	<3%	2–20	3–20%	>20	>20%	>20	>20%
**2019**	<2	<3%	2–20	3–20%	>20	>20%	>20	>20%

pNET, pancreatic neuroendocrine tumor; pNEC, pancreatic neuroendocrine carcinoma; HPF, high-power field; NA, not applicable.

**Table 2:** Staging of pNENs: AJCC 8th edition/ENETS staging.

AJCC 8th Edition and ENETS Staging for pNENs			
Stage	T *	N **	M
**I**	T1	N0	M0
**II(A) ^#^**	T2	N0	M0
**II(B) ^#^**	T3	N0	M0
**III(A) ^#^**	T4	N0	M0
**III(B) ^#^**	Any T	N1	M0
**IV**	Any T	Any N	M1

AJCC, American Joint Committee on Cancer; ENETS, European Neuroendocrine Tumor Society. * T stage: T1, tumor limited to the pancreas (<2 cm); T2, tumor limited to the pancreas (2–4 cm); T3, tumor limited to the pancreas (>4 cm) or invading the duodenum or common bile duct; T4, tumor invading the adjacent structures. ** N stage: N0, no regional lymph node metastasis; N1, regional lymph node metastasis. **^#^** Stage II (A/B) and III (A/B) are only used in the ENETS system.

## Data Availability

The data presented in this study are available in the present manuscript.
